# Early Achilles Enthesis Involvement in a Murine Model of Spondyloarthropathy: Morphological Imaging with Ultrashort Echo-Time Sequences and Ultrasmall Superparamagnetic Iron Oxide (USPIO) Particle Evaluation in Macrophagic Detection

**DOI:** 10.1155/2019/2834273

**Published:** 2019-03-28

**Authors:** Benjamin Dallaudiere, Aurelien J. Trotier, Emeline J. Ribot, Stéphane Loubrie, Sylvain Miraux, Olivier Hauger

**Affiliations:** ^1^Department of Radiology, University Hospital of Bordeaux, Bordeaux, France; ^2^Centre de Résonance Magnétique des Systémes Biologiques, UMR 5536, CNRS, University of Bordeaux, Bordeaux, France

## Abstract

**Purpose:**

To confirm the interest of 3-dimensional ultrashort echo-time (3D-UTE) sequences to assess morphologic aspects in normal and pathological Achilles entheses in a rat model of spondyloarthropathy (SpA) with histological correlations, in comparison with conventional RARE T2 Fat-Sat sequences, and, furthermore, to evaluate the feasibility of a 3D multiecho UTE sequence performed before and after the intravenous injection of ultrasmall superparamagnetic iron oxide (USPIO) particles to assess macrophagic involvement in the Achilles enthesis in the same rat model of SpA.

**Materials and Methods:**

Fourteen rats underwent in vivo MRI of the ankle at 4.7 T, including a 3D RARE T2 Fat-Sat sequence and a 3D ultrashort echo-time (UTE) sequence for morphologic assessment at baseline and day 3 after induction of an SpA model, leading to Achilles enthesopathy in the left paw (right paw serving as a control). A 3D multiecho UTE sequence was also performed at day 3 before and then 24 (4 rats) and 48 (2 rats) hours after intravenous injection of USPIO. Visual analysis and signal intensity measurements of all images were performed at different locations of the Achilles enthesis and preinsertional area. Visual analysis and T2∗ measurements were performed before and after USPIO injection, on the 3D multiecho UTE sequence in the same locations. Normal and pathological values were compared by Wilcoxon signed-rank tests. MR findings were compared against histological data.

**Results:**

3D-UTE sequences enabled morphologic identification of the anterior fibrocartilage and posterior collagenic areas of the Achilles enthesis. Visual analysis and signal intensity measurements distinguished SpA-affected entheses from healthy ones at day 3 (*P*=0.02). After administration of USPIO, no differences in signals were detected. Similarly, both visual analysis and signal T2∗ measurements in the enthesis were unable to distinguish the SpA-affected tendons from healthy ones (*P*=0.914). Neither the normal anatomy of the enthesis nor its pathological pattern could be distinguished using the standard RARE sequence. Histology confirmed the absence of USPIO in Achilles entheses, despite marked signs of inflammation.

**Conclusion:**

Unlike conventional RARE T2 Fat-Sat sequences, 3D-UTE sequences enable morphologic assessment of normal enthesis anatomy and early detection of abnormalities in pathological conditions. However, 3D multiecho UTE sequences combined with USPIO injections with T2∗ measurements were unable to detect macrophagic involvement in these pathological conditions.

## 1. Introduction

The human body contains many tissue components, mostly located in musculoskeletal organs such as tendons, with short T2 (transversal relaxation times). These are not, or only poorly, detected with conventional T2-weighted MR sequences [1–3]. This represents a limit of MR in early diagnosis of several pathologies, such as inflammatory enthesopathies with T2 tissues shorter than 10 ms [4]. The Achilles enthesis is composed of two main portions: an anterior fibrocartilaginous portion (from proximal to distal: periosto-fibrocartilage, sesamoido-fibrocartilage, and entheso-fibrocartilage) and a posterior collagenic portion with a posterior enthesis of collagenic fibrous tissue [5–7]. This enthesis is avascular in physiological conditions. In case of spondyloarthropathy (SpA) disorders, the anterior fibrocartilaginous is the target of an autoimmune process leading to a macrophagic and lymphoplasmocytic proliferation with a spared posterior collagenic portion [7].

Ultrashort echo-time (UTE) sequences have recently shown promising results to overcome the limitations of standard T2 sequences for murine Achilles tendon assessment. UTE sequences were able to highlight the “zonal” enthesis anatomy of the Achilles tendon and detect abnormalities very early in the course of an SpA-induced model with scan times (63 minutes to 12 hours) hardly compatible with clinical applications [8]. Ultrasmall superparamagnetic iron oxide (USPIO) particle-enhanced MRI has been widely used for inflammation process detection and evaluation [9, 10]. Following i.v. injection, USPIO is incorporated into macrophages via endocytosis. The uptake of USPIOs by phagocytic monocytes and macrophages provides an in vivo tool by which MRI can sensitize and monitor the involvement of macrophages in inflammatory processes, such as multiple sclerosis, stroke, brain tumors, nephropathies, and vulnerable plaques in the carotid artery [11–13]. To our knowledge, no study has evaluated the use of USPIO in tendon inflammation detection, despite the fact that gadolinium, the only contrast agent used in evaluation of tendinopathy, lacks both sensitivity (only advanced lesions are detected) and specificity (does not differentiate between mechanical and inflammatory lesions), leading to significant delays in diagnosis and implementation of the adopted treatment.

The objective of our study was threefold:

1. To confirm the interest of 3-dimensional ultrashort echo-time (3D-UTE) sequences to assess morphologic aspects in normal and pathological Achilles entheses, at 4.7 T, in a rat model of SpA with histological correlation in comparison with a conventional RARE T2 Fat-Sat sequence
2. To evaluate the feasibility of a 3D multiecho UTE sequence performed before and after the i.v. injection of USPIO to assess macrophagic involvement in the Achilles enthesis in the same rat model of SpA at 4.7 T
3. To demonstrate that the measurement of the T2∗ of the enthesis can be an early quantitative signal biomarker of SpA enthesopathy compared to a conventional sequence and a 3D-UTE sequence

## 2. Materials and Methods

### 2.1. Animal Model

Procedures and animal care complied with the “Principles of Animal Care” of the European Union, and animal experimentation was carried out under the authorization of the Ministry of Agriculture.

The study included 14 immunocompetent male Wistar rats (age = 10 weeks; mean weight = 252 g ± 32; Janvier Labs). All left paws were induced with a SpA-like Achilles tendon enthesopathy by subcutaneous injection of the Freund adjuvant as previously described [8, 14]. The right paws served as controls.

The rats were sedated before and during each manipulation with isoflurane (5% for induction and 2.5% for maintenance). They were housed in groups of 4 in stalling cages in a conventional animal housing facility, with a 12 h light/dark cycle (12 h light/12 h dark) at 20 ± 2°C temperature. They were given access to standard laboratory rat pellets with water ad libitum.

### 2.2. Contrast USPIO Agent

USPIO were synthesized in the Department of General, Organic and Biomedical Chemistry, NMR and Molecular Imaging Laboratory, University of Mons, Belgium. These nanosystems are based on small iron oxide cores surrounded by a thin polysiloxane shell exhibiting carboxylic acid functions (TEPSA-Rh-PEG NPs).

The mean core diameter was evaluated to be 7.8 nm. 3-(Triethoxysilyl)propylsuccinic anhydride (TEPSA) was used for coating. The global diameter was then evaluated as 17.66 nm. To ensure the colloidal stability of the nanosystems, the use of organofunctional silanes has been preferred to other more conventional polymeric matrices. Relaxivity was *r*1 = 22 mM^−1^·s^1^ at 1.5 T [15]. Before i.v. injection, USPIO endocytosis by microglia was tested *in vitro* (Supplementary Materials (available here)). Intravenous injection was performed via the tail vein, immediately after the baseline magnetic resonance scan, by one of the authors (ER) with 6 years of animal handling experience.

### 2.3. MR Imaging and Data Analysis

In vivo imaging sessions of the Achilles tendon from the myotendinous junction to the calcaneus enthesis were performed on a 4.7 tesla Bruker BioSpec system (Ettlingen, Germany) whose characteristics have already been described [8].

Rats were placed in the supine position with the straight leg and ankle at 90°. 3D RARE T2 Fat-Sat, 3D ultrashort echo-time (3D-UTE), and 3D multiecho UTE sequences without and with USPIO were performed.

3D RARE T2 Fat-Sat was used instead of a conventional long T2 sequence (typically 2D T2 fast spin echo) commonly used in human musculoskeletal imaging because of the high magnetic field used and the necessity of a very high spatial resolution in our animal model. The parameters were as follows: TR/TE = 1600/35 ms, FOV = 20 × 15 × 10 mm, matrix = 192 × 128 × 32, spatial resolution = 0.104 × 0.117 × 0.313 mm anisotropic, number of excitations = 2, reception bandwidth = 71 kHz, selective RF pulse = Shinnar–Le Roux (SLR) of 2 ms, inversion RF pulse = SLR of 1.7 ms, and acquisition time = 8 min 24 sec.

3D-UTE was used for morphological imaging as a very short TE sequence to assess tissue components in the Achilles enthesis with short T2 in normal and previous inflammatory SpA enthesopathies. The parameters were as follows: TR/TE = 11.863/0.031 ms, FOV = 17 × 17 × 17 mm, matrix = 128 × 128 × 128, spatial resolution = 0.133 mm isotropic, number of excitations = 3, spokes = 51360, reception bandwidth = 100 kHz, flip angle = 10.0°, square excitation pulse = 0.05 ms, fat saturation module = pulse (bandwidth 701.19 Hz, duration 7.981 ms, and angle 90°), and total acquisition time = 30.5 min. The Fat-Sat pulse was applied for each TR. These sequences were performed on 14 rats at baseline (D0) and at preclinical SpA phase 3 days after the induction of the disease (D3) for morphologic assessment.

The 3D multiecho UTE sequence, performed before and after USPIO injection, allowed T2∗ measurements as an early signal biomarker for SpA enthesopathy, in comparison with conventional 3D RARE T2 Fat-Sat and 3D-UTE sequences. Multiple echoes were acquired at 8 TE values: 0.031/0.1/0.2/0.3/0.5/0.8/1/1.2 ms to quantify the T2∗ of the tendon. The other parameters were as follows: TR = 4 ms, FOV = 20 × 20 × 20 mm, matrix = 128 × 128 × 128, spatial resolution = 0.156 × 0.156 × 0.156 mm, number of excitations = 1, spokes = 51360, bandwidth = 100 kHz, flip angle = 8.0°, block pulse excitation, and total acquisition time = 27 min. No fat saturation was applied.

This sequence was performed on 4 rats before (day 3 after the induction of the disease) and 24 hours after i.v. injection of 200 *µ*mol/kg (2 rats) and 600 *µ*mol/kg (2 rats) of USPIO.

The population injected with 200 *µ*mol/kg (2 rats) was sacrificed subsequently (day 4). The population injected with 600 *µ*mol/kg (2 rats) was imaged again 48 hours after contrast injection (day 5) before sacrifice for histologic analysis.

After the in vivo experiments, the paw specimens (distal portion of the leg with ankle and foot with intact soft tissues) were removed from the animal body and incubated with 4% paraformaldehyde (PFA) overnight for tissue fixation. They were then placed, with ankle at 90°, in a tube containing 15 ml 70% ethanol. All rats were sacrificed at D5 for histological analysis.

Qualitative analysis and signal intensity measurements were performed on all sequences by a senior musculoskeletal radiologist.

### 2.4. Morphologic Evaluation

All images were analyzed using the MATLAB-based software (MATLAB®). Signal intensity measurements were performed on in vivo RARE T2 Fat-Sat images and 3D-UTE images in the axial plane of the tendon, to better distinguish the enthesis fibrocartilage located anterior from the collagenic portion located posteriorly for the 14 rats. Four regions of interest (ROIs) were placed in the enthesis: one (1 mm^2^) in the anterior fibrocartilage, one (1 mm^2^) in the posterior collagenic portion, one (3 mm^2^) covering the entire zone, and a fourth one in the subcutaneous fat. The same procedure was performed on the (entirely collagenic) preinsertional tendon, located just proximal to the enthesis, considered as a control. Results were expressed as normalized signal-to-noise ratio (nSNR) which is the standard signal-to-noise ratio (SNR) normalized to the subcutaneous fat signal measured at the same level using the following formula: nSNR = SNR/signal (fat tissues) = signal (tendon tissue)/(SD (noise) ∗ signal (fat tissue)).

The Gaussian noise standard deviation, SD (noise), was calculated as the standard deviation of the signal measured in a background ROI on magnitude image, divided by 0.695. This value is used to correct the Rayleigh distribution of noise for a four-element phase-array coil, as shown by Constantinides et al. [16].

### 2.5. Signal Evaluation

T2∗ measurements were performed on in vivo 3D multiecho UTE images, before and after the injection of USPIO, in the axial plane of the tendon on 4 rats. An ROI was placed in the enthesis (3 mm^2^) and the preinsertional tendon and covered the entire zone. The results were expressed as mean ± standard deviation of the measurements. T2∗ values were acquired by direct ROI signal measurements, with no consideration of the noise. The fit model was expressed as *B* × exp(*t*/T2∗).

Statistical analysis was then carried out with SAS software, Version 9.4 (SAS Institute Inc.). nSNR and T2∗ data, because of their small number, were not normally distributed. Therefore, we used a nonparametric test (Wilcoxon signed-rank test) to compare in vivo data obtained from all sequences at different locations of Achilles tendons at different time points.

### 2.6. Tendon Pathology

The paws submitted to *in vivo* imaging were embedded in paraffin, cut into 5 *µ*m sagittal slices, and stained:

- Hematoxylin and eosin (HE) and Masson's trichrome (MT) stain was used for morphologic assessment. Enthesis and preinsertional tendons were assessed for the presence and/or aspects of tenocytes, ground substance, collagen, and vascularity [17, 18].
- Perls' coloration (PC) was assessed for USPIO detection. This coloration shows the insoluble iron in the form of blue-green grains in the cytoplasm of cells, i.e., macrophages.
- Immunolabeling using ED1 mouse monoclonal antibodies specific for rat macrophages was also used to demonstrate macrophages in the tendons and determine their distribution in different tendon areas. The antibody was incubated for 12 h at 4°C at a dilution of 1/1000. Revelation was made through DAB (3,3′-tetrahydrochloride diaminobenzidine) staining after an incubation of biotinylated anti-rat IgG.

## 3. Results

### 3.1. Animals

From day (D) 0 to day 5, all animals were asymptomatic without evidence of Achilles tendon soreness. All rats survived the Freund adjuvant and the USPIO injections.

### 3.2. MR Imaging and Data Analysis

#### 3.2.1. Morphologic Evaluation

nSNR measurements (normalized with subcutaneous fat signals at the same level) of the enthesis and preinsertional tendons measured in vivo on both control and pathologic groups (at D0 and D3) are summarized in Tables 1 and 2.

**Figure 1:**
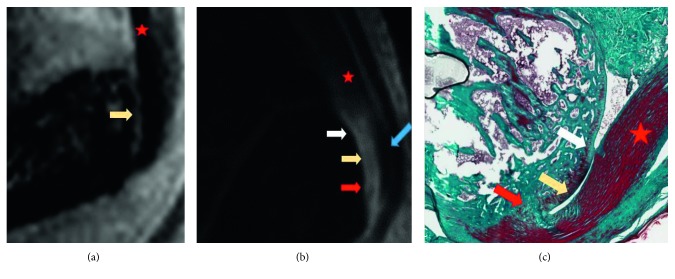
Sagittal RARE T2 Fat-Sat sequence (a) and 3D-UTE PD-w acquisition sequence (b) with focus on normal enthesis area (in vivo) for morphologic assessment. The anterior part of the enthesis looks brighter than the posterior part in in vivo assessment in UTE sequences (b) but not in RARE T2 Fat-Sat acquisition sequences (a) at D0. Correlation with histology in the same normal rat in MT (c). Red star: enthesis in a preinsertional tendon; yellow arrow: sesamoido-fibrocartilage; white arrow: periosto-fibrocartilage; red arrow: entheso-fibrocartilage; blue arrow: fibrous enthesis in a normal Achilles tendon. MT: Masson's trichroma; PD-w: proton density weighted images.

  Tendons at baseline (day 0):

- 3D RARE images: On visual analysis, both preinsertional tendons and entheses appeared of uniformly low signal and were indistinguishable from one another, without any possibility to define the intrinsic anatomy of the enthesis. This was confirmed on quantitative analysis as no significant signal intensity (SI) differences were observed on ROI measurements of preinsertional tendons and entheses (Figure 1(a)).
- 3D-UTE images: On visual analysis, the preinsertional tendon appeared of uniformly low signal. The enthesis appeared globally brighter than the preinsertional tendon. Two portions of the enthesis could be differentiated: an anterior portion of high signal and a posterior portion of lower signal. This was confirmed on quantitative analysis, with the global SI in the enthesis at 12.6 versus 3.5 in the preinsertional tendon (*P*=0.0006) and SI in the anterior portion of the enthesis at 13.2 versus 8.3 (*P*=0.016) in the posterior part (Figure 1(b)).

**Figure 2:**
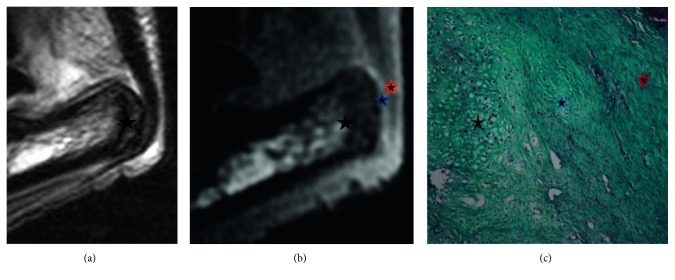
Sagittal RARE T2 Fat-Sat sequence (a) and 3D-UTE PD-w acquisition sequence (b) with focus on normal preinsertional and pathological enthesis area (in vivo) for morphologic assessment. The anterior part of the enthesis looks brighter than the posterior part in in vivo assessment in UTE sequences but not in RARE T2 Fat-Sat acquisition sequences at D3. Correlation with histology in the same rat before USPIO injection in MT (c) with moderate fibrillar disorganization of the anterior fibrocartilage (blue star) and normal posterior enthesis (red star). Calcaneum bone marrow is represented by black star.

  Tendons at day 3:

- 3D RARE images: On visual analysis, both preinsertional tendons and entheses in SpA-induced animals remained of uniformly low signal and were still indistinguishable from one another. This was confirmed on quantitative analysis as no significant signal intensity (SI) differences were observed on ROI measurements of preinsertional tendons and entheses (Figure 2(a)).
- 3D-UTE images: In the pathologic paws, by visual analysis, the preinsertional tendon appeared of uniformly low signal. The enthesis appeared with a signal much higher than that of the preinsertional tendon, and the two inner portions were still differentiable. This was confirmed on quantitative analysis as global SI in the enthesis was 16.5 versus 3.6 in the preinsertional tendon (*P*=0.0037) and SI in the anterior portion of the enthesis was 25.6 versus 15.9 (*P*=0.0057) in the posterior part. Moreover, the enthesis at day 3 was significantly brighter than that at baseline (16.5 vs. 12.6; *P*=0.025) (Figure 2(b)).

#### 3.2.2. Signal Evaluation

T2∗ measurements (*µ*s) of the entheses and preinsertional tendons measured *in vivo* on both control and pathologic groups (at D3 and D4) are summarized in Table 3.

**Figure 3:**
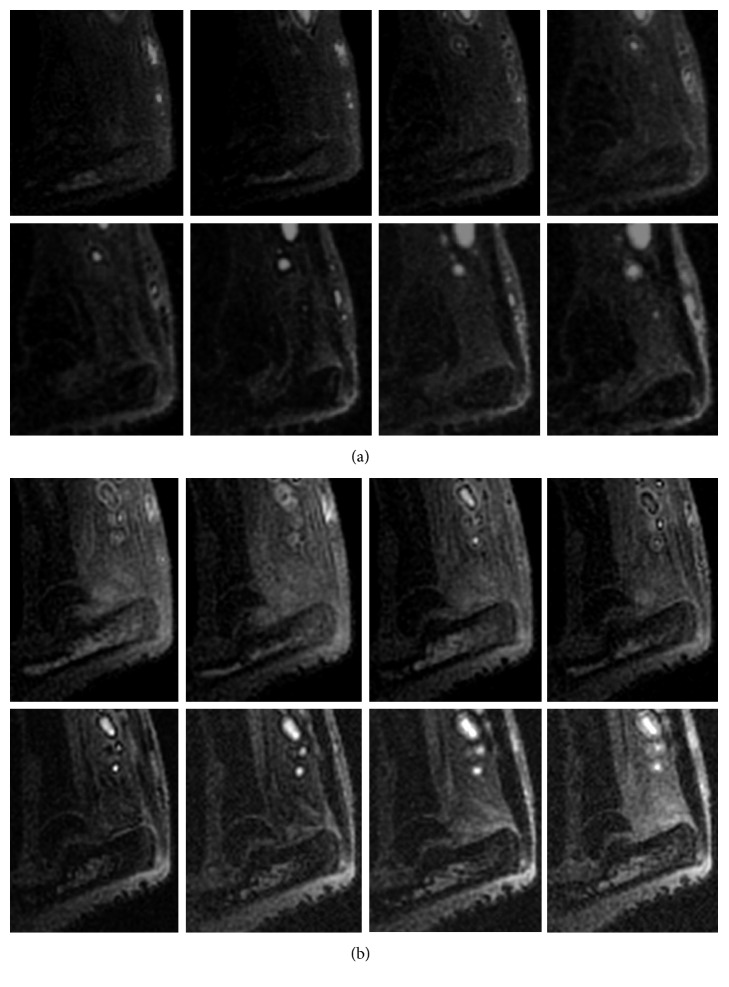
(a) Sagittal in vivo 3D multiecho UTE images, at D3 (8 TE values: (A) 0.031, (B) 0.1, (C), 0.2, (D) 0.3, (E) 0.5, (F) 0.8, (G) 1, and (H) 1.2 ms) before USPIO injection, with focus on enthesis pathological area signal assessment for the same rat. (b) Sagittal in vivo 3D multiecho UTE images 24 hours later, at D4 (8 TE values: (A) 0.031, (B) 0.1, (C) 0.2, (D) 0.3, (E) 0.5, (F) 0.8, (G) 1, and (H) 1.2 ms) after USPIO injection, with focus on enthesis pathological area signal assessment for the same rat. No signal drop was noted after USPIO injection in 3D multiecho UTE images.

*(i) Tendons at Day 3 (before USPIO Injection)*. On visual analysis of 3D multiecho UTE images, the preinsertional tendon appeared of uniformly low signal. The enthesis appeared globally brighter than the preinsertional tendon, and two portions could be differentiated: an anterior portion of high signal and a posterior portion of lower signal. No significant differences were observed on T2∗ measurements between preinsertional tendons and entheses (*P*=0.68 and *P*=0.14 for the healthy and the SpA paws, respectively) (Figure 3(a)).

*(ii) Tendons at Days 4 and 5*. At day 4, no signal drop was visually observed after USPIO injection (whether 200 or 600 *µ*mol/kg). This was confirmed on quantitative analysis as the global T2∗ measurement in the enthesis was 0.41 before and after USPIO injection (*P*=0.91). Similarly, in the preinsertional tendon, the T2∗ values were 0.32 and 0.45 before and after USPIO injection, respectively (*P*=0.32). Moreover, the T2∗ measurements between the enthesis and the preinsertional tendon were not significantly different (*P*=0.15 and 0.49 for the healthy and the SpA paws, respectively) (Figure 3(b)).

At day 5, no signal drop was visually observed after USPIO injection. This was also confirmed on quantitative analysis with no significant enthesis T2∗ measurements before and after USPIO injection (*P*=0.62). Similarly, in the preinsertional tendon, the T2∗ values were not statistically different before and after USPIO injection (*P*=0.65).

#### 3.2.3. Tendon Pathology

Histology could individualize the two portions of the enthesis (anterior fibrocartilage and posterior collagenic portions) in all rats. Moreover, the different parts of the anterior fibrocartilage, i.e., sesamoido-fibrocartilage, entheso-fibrocartilage, and periosto-fibrocartilage, could be identified (Figure 1(c)).

**Figure 4:**
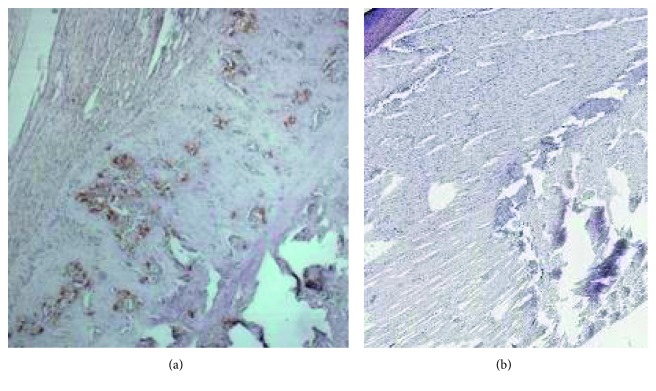
(a) Positive ED1 immunohistochemistry (magnification 200x) in SpA enthesitis in the previous phase. Tissue showing brown macrophages, confirming the efficiency of the model. (b) Negative Perls' coloration (magnification 200x) in SpA enthesitis in the previous phase in the same rat. Tissue is without blue-green granules (ferric ferrocyanide).

Negative immunostaining on HE and MT confirmed the absence of abnormality at baseline in every part of the tendon, especially the preinsertional portion and the enthesis. At day 3 after induction of the disease, all 14 rats showed moderate fibrillar disorganization of the anterior fibrocartilage (specifically the sesamoido-fibrocartilage), confirming the development of the disease (Figure 2(c)) with moderate macrophagic infiltration on ED1 staining (Figure 4(a)). Perls' coloration was negative in all 4 USPIO-injected rats, confirming the absence of iron (Figure 4(b)).

## 4. Discussion

Three main results can be drawn from this study. First, we confirmed the recently published data showing the efficiency of 3D-UTE sequences, compared to standard (RARE T2 Fat-Sat) sequences, in determining the morphologic intrinsic normal anatomy of the Achilles enthesis in a murine model. We were able to distinguish in all rats, both visually and quantitatively, the anterior cartilaginous part from the posterior collagenic portion and then to highlight the “zonal” enthesis anatomy of Achilles tendons with histologic confirmation. However, compared to the previous study, in the present study, we obtained similar results with half the scan time (30 vs. 63 min) and with minimal spatial resolution loss (0.133 vs. 0.106 mm), which is of paramount importance for clinical applications [8].

Second, the 3D-UTE sequence, contrary to the routinely used RARE T2 Fat-Sat sequence, can detect morphologic abnormalities very early in the course of the SpA-induced model, in agreement with histological data. This is of paramount importance in pathological conditions as tendons, or more particularly tendon entheses, are the initial and main target of inflammatory diseases such as spondyloarthropathies in the form of a lymphoplasmocytic proliferation initially located in the entheso-fibrocartilage [19–21].

Last, 3D multiecho UTE sequence combined with USPIO injection and consequently the T2∗ values did not vary in pathological conditions. Indeed, while we found typical morphological features of SpA enthesitis using UTE imaging, USPIO did not accumulate in our murine model with SpA, despite moderate macrophagic involvement on histology. This surprising result raises different points of discussion concerning the quality of injection, the physics properties of the USPIO used in the study, the dose, or the postinjection imaging delay.

The i.v. injection was directly injected to the tail of the rats by a very experienced operator with 6 years of animal handling experience. Moreover, before i.v. injection, USPIO endocytosis was positively tested in vitro (additional material). The physical properties of our TEPSA-Rh-PEG NPs are comparable to those of USPIO particles used in the scientific literature in terms of size (17.66 nm), relaxivity (*r*1 = 22 mM^−1^·sec^−1^ at 1.5 T), and observation of their *in vivo* behavior in murine blood circulation. P904® and ferumoxytol (Feraheme TM) are USPIO composed of a nonstoichiometric magnetite core surrounded by a polyglucose sorbitol carboxymethylether coat with a diameter of 25–30 nm, a 750 kDa molecular weight, and 145 min blood half-life (with 200 *µ*mol/kg). Concerning ferumoxytol, the relaxivity constant at 2 T MRI field is 58.609 mM^−1^·sec^−1^. However, in our opinion, there are no obvious physical properties of our contrast agent that could explain the absence of accumulation in the tendon [15].

The signal drop T2∗ data are dose dependent [10–12]. In our study, a standard dose of 200 *µ*mol/kg was first injected in two rats followed by a dose of 600 *µ*mol/kg that represents an important concentration of 3 times the standard dose. Higher concentrations could not be injected because of blood thrombus risk. Therefore, the dose is probably not an explanation for the absence of iron within the tendon, as is the postinjection delay, since 3D multiecho UTE sequences were performed 24 h but also 48 h after USPIO injection.

As a result, we may consider two options explaining the absence of USPIO particles within the SpA Achilles tendons. (i) An insufficient number of macrophages for significant iron uptake and (ii) an insufficient vascular supply of the enthesis at this very early stage of the disease lead to limited UPSIO particle distribution.

Until now, the scientific literature investigating the use of USPIO in tendon pathologies has focused on i.v. injection of USPIO-labeled mesenchymal stem cells to achieve tendon healing, which differs significantly from our direct i.v. particle injection approach. Scharf et al. injected USPIO-labeled mesenchymal stem cells into an ovine model of mechanical or chemical tendonitis [22]. This study provided promising results, indicating this to be an effective method for cell tracking in a large animal model of tendon injury for up to 7 days after injection. However, tendons were strictly scanned ex vivo at 7 and 14 days to determine the presence and distribution of intralesional cells with no in vivo MRI assessment [22]. In the same way, Yang et al. [23] demonstrated on a rabbit model that USPIO is suitable to label tendon stem cells and track them in vitro and in vivo using MR imaging, which simultaneously offers a noninvasive method to monitor repair of injured tendons. Castaneda et al. also described efficient labeling of three kinds of stem cells with ferumoxytol that led to significant MR signal effects. This technique may be applied for noninvasive monitoring of stem cell therapies in preclinical and clinical settings [24].

Our study has some limitations. The first concerns the animal model and injection method of the Freund adjuvant. This model, known to be very specific for spondyloarthropathy, is however the only nonautoimmune model validated in the literature [14]. Moreover, the strong homogeneity and reproducibility of the morphologic data obtained in different parts of the tendons on both sequences suggest the absence of spreading.

The relatively small number of animals is also a limitation, but quantitative data were sufficiently homogeneous to lead to high levels of statistical significance in the results. To compare with 3D-UTE sequences, we chose a long 3D RARE T2 sequence instead of the more conventional T2 sequence (typically 2D T2 fast spin echo) commonly used in human musculoskeletal imaging because of the high magnetic field used and the need for a very high spatial resolution in our animal model. Last, the UPSIO-injected sample size was small but sufficient to show the absence of interest of USPIO in this model of SpA-induced Achilles enthesitis.

Finally, it is unclear to what extent the results of this animal study can be extrapolated to humans. However, as the anatomy and types of lesions of the tendons were radiologically and histologically similar, these results can serve as a good reference to what could be expected in humans. A similar study on a human population will be necessary to confirm these results.

In conclusion, unlike RARE T2 Fat-Sat sequences, 3D-UTE sequences enable morphologic assessment and visualization of normal enthesis anatomy with early detection of abnormalities in pathological conditions compared to histological data. However, the presence of (a limited number of) macrophages in the pathologic tendons could not be detected by i.v. injection of USPIO particles on the 3D multiecho UTE sequence combined with T2∗ measurements.

## Figures and Tables

**Table 1:** Normalized signal-to-noise ratio (nSNR) (normalized to the subcutaneous fat signal at the same level) of different parts of the rat tendon calculated from the in vivo RARE images.

RARE	Preinsertional area			Enthesis		
	Anterior	Posterior	Global	Anterior	Posterior	Global
D0	0.62 ± 0.08	0.65 ± 0.05	0.63 ± 0.12	0.73 ± 0.06	0.67 ± 0.06	0.68 ± 0.1
D3	0.72 ± 0.08	0.65 ± 0.05	0.8 ± 0.1	0.64 ± 0.04	0.6 ± 0.26	0.7 ± 0.1

D: day.

**Table 2:** Normalized signal-to-noise ratio (nSNR) (normalized to the subcutaneous fat signal at the same level) of different parts of the rat tendon calculated from the in vivo UTE images.

UTE	Preinsertional area			Enthesis		
	Anterior	Posterior	Global	Anterior	Posterior	Global
D0	4.3 ± 0.61	3.77 ± 0.25	3.53 ± 0.25	13.2 ± 1.7^∗^	8.27 ± 0.64	12.63 ± 0.55
D3	5.07 ± 1	3.6 ± 0.4	3.63 ± 0.32	25.6 ± 1.22^∗^	15.87 ± 0.12	16.47 ± 1.17

Unlike the conventional RARE T2 sequence, the 3D-UTE sequence enables visualization of the enthesis anatomy (^∗^) at D0 and D3 (*P* < 0.024) but also early detection of abnormalities in pathological conditions at D6. D: day.

**Table 3:** T2∗ (*µ*s) of different parts of the rat tendon calculated from the 3D multiecho UTE images, in preclinical SpA with 600 *µ*mol/kg injection.

T2∗ (*µ*s)	Preinsertional area	Enthesis
Healthy tendon, before USPIO injection at D3	374.7 ± 29.5	408.3 ± 57.3
Healthy tendon, after USPIO injection at D4	411.1 ± 6.3	432 ± 24.3
Pathological tendon, before USPIO injection at D3	323.8 ± 42.4	405.1 ± 15.6
Pathological tendon, after USPIO injection at D4	452 ± 142.3	411.9 ± 85.9

3D multiecho UTE sequence combined with USPIO injection did not detect abnormalities in pathological conditions. D: day.

## Data Availability

The data used to support the findings of this study are available from the corresponding author upon request.

## Supplementary Materials

Supplementary Materials Before i.v. injection, USPIO endocytosis was in vitro tested and positive to avoid absence of endocytosis.
