# Supplementary material for: Early Achilles Enthesis Involvement in a Murine Model of Spondyloarthropathy: Morphological Imaging with Ultrashort Echo-Time Sequences and Ultrasmall Superparamagnetic Iron Oxide (USPIO) Particle Evaluation in Macrophagic Detection
Source: Contrast Media Mol Imaging. 2019 Mar 28;2019:2834273. doi: 10.1155/2019/2834273 (PMC6458856; doi:10.1155/2019/2834273)

Supplementary material:

Before i.v. injection, USPIO endocytosis was *in vitro* tested and positive to avoid absence of endocytosis.

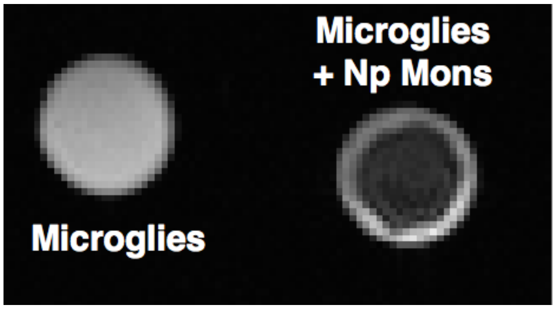

Supplement: Supplementary Materials — Before i.v. injection, USPIO endocytosis was in vitro tested and positive to avoid absence of endocytosis. [file 2834273.f1.pdf]
